# Drought stress-induced the formation of heteromorphic leaves of *Populus euphratica* Oliv: evidence from gene transcriptome

**DOI:** 10.3389/fpls.2023.1194169

**Published:** 2023-06-07

**Authors:** Rui Xu, Wei-Guo Liu, Ting-Wen Huang, Bo-Rui Li, Hui-Xian Dai, Xiao-Dong Yang

**Affiliations:** ^1^ College of Ecology and Environment, Xinjiang University, Urumqi, China; ^2^ Key Laboratory of Oasis Ecology of Education Ministry, Xinjiang University, Urumqi, China; ^3^ Department of Geography and Spatial Information Techniques/Center for Land and Marine Spatial Utilization and Governance Research, Ningbo University, Ningbo, China

**Keywords:** arid desert, drought stress, heteromorphic leaves, genetic expression, RNA-seq

## Abstract

*Populus euphratica* Oliv., a dominant species of arid desert community, grows heteromorphic leaves at different crown positions. Whether heteromorphic leaves are a strategy of plant adaptation to drought stress is rarely reported. This study sequenced the transcriptome of three typical heteromorphic leaves (lanceolate, ovate and broad-ovate leaves) of *P. euphratica*, and measured their drought stress. We wanted to reveal the molecular mechanisms underlying the formation of heteromorphic leaves. Drought stress was increased significantly from lanceolate to ovate to broad-ovate leaves. Gene ontology (GO) and KEGG enrichment analysis showed that the MADs-box gene regulated the expression of peroxidase (POD) in the phenylpropane biosynthetic pathway. The up-regulated expression of the chalcone synthase (CHS) gene in broad-ovate leaves significantly activated the flavonoid biosynthetic pathway. In the process of leaf shape change, the different expressions of homeodomain leucine zipper (HD-ZIP) among the three heteromorphic leaves had potential interactions on the AUX and ABA pathways. The expression of Sucrose phosphate synthase (SPS) and sucrose synthase (SUS) increased from lanceolate to broad-ovate leaves, resulting in a consistent change in starch and sucrose content. We concluded that these resistance-related pathways are expressed in parallel with leaf formation genes, thereby inducing the formation of heteromorphic leaves. Our work provided a new insights for desert plants to adapt to drought stress.

## 1 Introduction

Leaves are very important organs of plants because they determine photosynthesis, transpiration and photoreception (Coble et al., 2017; Wang and Jiao, 2020). At the community and ecosystem level, leaf traits, such as leaf area, specific leaf area, shape, and growth time, are closely related to ecosystem biomass and function through niche separation or overlap, thus affecting community stability (Sakschewski et al., 2015; Fan et al., 2022; Huang et al., 2022). As the most important adaptive strategy of plants to the environment, the shape of the leaves is not invariant, but changes with the environment. (Chitwood and Sinha, 2016; Gupta et al., 2019). For example, with the decrease of precipitation and temperature, or the increase of drought from low to high latitudes, leaves gradually changed from flat (i.e., round, fan, rhombic, oval and rhombus) to cylindrical shapes (i.e., needle) (Li et al., 2017; Horiguchi et al., 2019). However, existing studies have mainly focused on revealing the underlying causes of differences in leaf shape between species, and the variation of leaf shape of the same species on a large environmental gradient, such as temperature, precipitation, and drought (McKee et al., 2019; Costa e Silva et al., 2021; Vento et al., 2022). In contrast, few studies have addressed changes in leaf shape within a plant individual.

One of the main causes of little fruitful attention to changes in leaf shape within the same plant individual is genetic limitation (Kessler and Sinha, 2004). In nature, the leaf shape of most plants does not change in the developing process from young to mature leaves, whereas the area increases in equal proportion (Pervaiz et al., 2022). The reason is that plants are genetically restricted to form a more stable structure to adapt the environmental conditions after long-term evolution, which can ensure that plants get the maximum benefit with the least investment (Wright et al., 2004). However, in variable or harsh environments, researchers have found a few species using the varied-shaped leaves to adapt to the environment and improve their survival rates. For example, emergent plants (such as *Batrachium bungei, Myriophyllum aquaticum, and Rotala rotundifolia*) living in the water-land transition zone, and *Cudrania tricuspidata* living on rocks of the coastal zone with soil drought and poor-soil nutrition, grow different shaped leaves on the same individual (Wersal and Madsen, 2011; Butkuvienė et al., 2020; Chu et al., 2021). Heteromorphic leaves refer to the phenomenon that plant individual grows different shaped leaves (e.g., lanceolate, ovate, and broad-ovate) at the different locations of the body (Iqbal et al., 2017; Zhai et al., 2020). The variety of leaf shapes ensures better adaptability and survival in variable environmental conditions (Kim et al., 2018). The existence of heteromorphic and normal (unchanged in shape) leaves may be a strategic tradeoff between “single and multipronged bets” (Sanginés de Cárcer et al., 2017; Pérez et al., 2021). In favorable and stable environments, plants tend to be stable in a particular way of life that maximizes their return on investment (single bet). On the contrary, When the environment changes or becomes harsh, the “multipronged bet” will give the plant a variety of options to survive, even if the benefits are not stable (Odening et al., 1974; Song et al., 2021). However, the current studies regarding of heteromorphic leaves mainly concentrated on the traits difference between the varied shaped-leaves (such as leaf area, thickness, specific leaf area, twig-leaf relationship) (Li et al., 2019; Wyka et al., 2019; Zhai et al., 2022), as well as the ecological effects of the shape change (Shabnam and Pardha-Saradhi, 2016). Few studies have revealed the mechanism of heteromorphic leaf formation from the perspective of molecular biology.

Water-land transition zone and extreme drought conditions have found to be the most likely habitats for heteromorphic leaf species (Li et al., 2017). The internal cause may be the formation of heteromorphic leaves determined by hydraulic constraints (Leigh et al., 2011). This is because plants in these areas are prone to hydraulic failure and drought-induced death due to the fact that they are suffered drought stress (Körner, 2019; Garcia et al., 2021). Yang et al. (2022a) has confirmed this speculation that, with the plant development, the progressive increases of gravitational potential between soil and plant canopy and the frictional resistance of water flow in xylem increases xylem tension, which in turn triggers hydraulics dysfunction through embolization. To reduce the effect of hydraulic constraints, plants adjust leaf morphology to improve hydraulic efficiency. As a result, plant develops different shaped leaves at different body locations. Studies have proved that heteromorphic leaves reflect the adaptability of plants to environmental changes through their phenotypic plasticity (Horiguchi et al., 2019; Wu et al., 2023). However, the relationship between plant drought stress and gene regulation inducing the formation of heteromorphic leaves has not been analyzed in previous studies. We have not yet known which genes and physiological processes regulate the formation of heteromorphic leaves through the gradient of drought stress.

*Populus euphratica* Oliv., a dominant species of arid desert riparian forest in Central and West Asia (Wang et al., 2018), is one of the few tree species in the terrestrial ecosystems that grows heteromorphic leaves at different positions (Li and Zheng, 2005; Zeng et al., 2019; Zhai et al., 2022). Revealing the relationship between drought stress and genetic expression in *P. euphratica* will help to understand the formation mechanism of heteromorphic leaves. However, most of the early studies on heteromorphic leaves of *P. euphratica* have described the differences in structure, physiological traits and protein expression of leaves with different shapes (Liu et al., 2015; Hao et al., 2017; Bao et al., 2019; Zhai et al., 2020), but little is known about the molecular regulatory mechanisms. In this study, we selected three typical heteromorphic leaves of *P. euphratica*, and then measured drought stress, and carried out transcriptome sequencing of different heteromorphic leaves. We set out to answer three scientific questions: (1) whether droughts stress was different among three heteromorphic leaves; (2) what are the genes and pathways involved in the formation of heteromorphic leaves? and (3) whether the changes in the expression of genes related to the formation of heteromorphic leaves are conducive to improving the leaf drought resistance.

## 2 Materials and methods

### 2.1 Experimental site

The experimental site is located in the Ebinur Lake Wetland Nature Reserve (82°36′–83°50′E, 44°30′–45°09′N) in the southern part of the Gurbantunggut Desert, Xinjiang Uygur Autonomous Region, in NW China. Affected by the typical temperate continental climate, the average annual air temperature ranges from 5 to 6°C, and the extreme maximum and minimum temperatures is 44°C and -33°C, respectively (Wang et al., 2021). The four seasons are distinct, with winter and summer significantly longer than spring and autumn. The annual precipitation in this area is unevenly distributed, mainly concentrated in summer. Mean annual precipitation is less than 100 mm, while annual potential evaporation ranges from 1500 to 2000 mm. Affected by sparse rainfall and the extremely arid climate, the zonal plant community is mainly composed of xerophyte desert plants (Zhang et al., 2018).

### 2.2 Plant material and sampling

**Figure 1:**
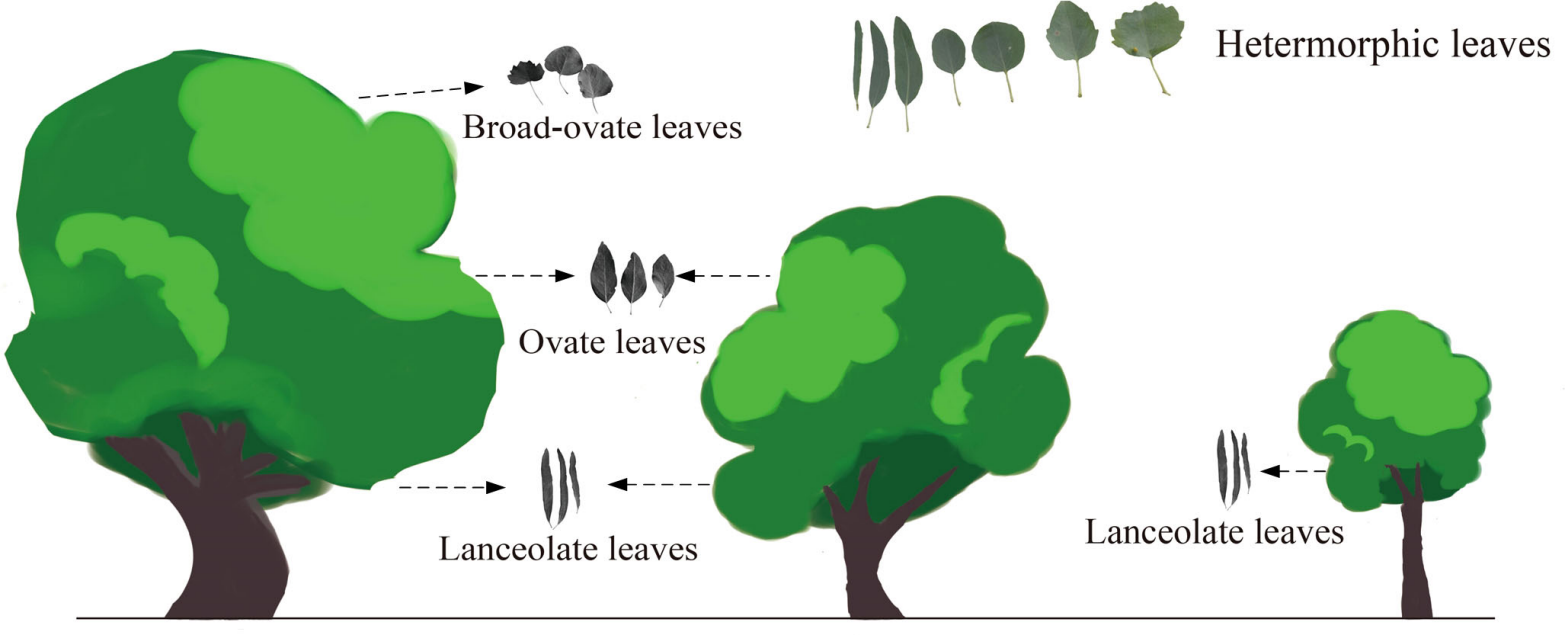
Spatial distribution of three types of heteromorphic leaves.

As suggested by Guo et al. (2020), the heteromorphic leaves can be categorized into three typical types based on morphological properties: lanceolate, ovate, and broad-ovate leaves. As the **Figure 1** suggested, lanceolate, ovate, and broad-ovate leaves grow different positions of *P. euphratica*. The lanceolate leaves distributed near the ground [i.e., all crown of the small trees (SLL) and the bottom of the medium (MLL) and large tree crowns (LLL)], while the ovate leaves distributed in the higher vertical position [(i.e., the top of the medium tree crowns (MOL) and the intermediate of the large tree crowns (LOL)], but the broad-ovate leaves only located at the top of the large tree crowns (LBL) (Hao et al., 2017).

In a 100 m × 100 m long-term dynamic observation and research field of *P. euphratica* community established by the Xinjiang University, nine *P. euphratica* individuals (three individuals for small, medium, and large trees, respectively) were randomly selected as sampling objects. At the leaf expansion stage of *P. euphratica* in mid-April 2019, the young leaves were randomly collected from each selected induvial, and then classified them according to the leaf shape. A total of 6 groups of leaf samples were collected, which included SLL, MLL, LLL, MOL, LOL and LBL. All samples were numbered and stored in liquid nitrogen tanks. After that, it was sent to the Novogene companies in the Beijing of China for RNA extraction and transcriptome sequencing.

### 2.3 RNA extraction, cDNA library construction, and clustering and sequencing

A total of six group samples were used for RNA-seq, and three replicates were performed for each group. RNA integrity and library quality was assessed using the RNA Nano 6000 Assay Kit of the Bioanalyzer 2100 system (Agilent Technologies, CA, USA), and the Agilent Bioanalyzer 2100 system, respectively. The index-coded samples were clustered on a cBot Cluster Generation System using TruSeq PE Cluster Kit v3-cBot-HS (Illumia) according to the manufacturer’s instructions. After cluster generation, library preparations were sequenced on an Illumina Novaseq platform. A total of 150 bp paired-end reads were generated.

### 2.4 Sequence assembly, annotation, and identification of the DEGs

Clean data (clean reads) were obtained by removing reads containing adapter, reads containing ploy-N and low-quality reads from raw data. At the same time, Q20, Q30 and GC content of the clean data were calculated. The reference genome index was built using Hisat2 v2.0.5 and paired-end clean reads were aligned to the reference genome using Hisat2 v2.0.5 (Kim et al., 2015). And then FPKM of each gene was calculated based on the length of the gene and read count mapped to this gene (Trapnell et al., 2010). Differential expression analysis of three conditions/groups (three biological replicates per condition) was performed using the DESeq2 R package (1.20.0) (Anders and Huber, 2010). Genes with an adjusted P-value <0.05 found by DESeq2 were assigned as differentially expressed.

### 2.5 GO and KEGG enrichment analysis of DEGs

Gene Ontology (GO) enrichment analysis of differentially expressed genes was implemented by the “clusterProfiler” package of R statistical software. GO terms with the corrected *p*-values < 0.05 were considered significantly enriched by differential expressed genes (Young et al., 2010). The statistical enrichment of differential expression genes in KEGG pathways were test using “clusterProfiler” package of R statistical software (Minoru et al., 2008).

### 2.6 Drought stress

Midday water potential and the contents of malondialdehyde (MDA), proline, and soluble sugar of leaves were used as the proxies to reflect the drought stress (Cao et al., 2020; Yang et al., 2022a). Since the young leaves collected in mid-April were too small to meet the experimental measurement requirements, we collected leaves from each selected individual in the peak season of plant growth (early July), and then measured the proxies of drought stress in the laboratory. To be specific, the heteromorphic leaves were collected from local 12:00 p.m. to 2:00 p.m, as suggested by Yang et al. (2022a). All samples were placed in ziplock bags, stored in a 0°C to 4°C refrigerator, and brought back to the laboratory. Then, the midday leaf water potential was measured with a Water Potential Meter (WP4-C, Degacon Devices Inc., Pullman, WA, USA). After that, as suggested by the methods of Li (2000), the refrigerated fresh leaves were taken out again and cut into small pieces. The contents of MDA, proline, and soluble sugar were measured by thiobarbituric acid colorimetry, sulfosalicylic acid colorimetry, and anthrone colorimetry, respectively. The measurements were repeated at least three times for each group of heteromorphic leaves.

## 3 Results

### 3.1 Difference in drought stress among different heteromorphic leaves

The contents of MDA, proline, and soluble sugar were increased significantly from lanceolate to ovate to broad-ovate leaves (*p*< 0.01), while midday leaf water potential showed an opposite pattern (*p*< 0.05). However, all proxies of drought stress were not changed significantly among the groups with the same leaf shape (i.e., SLL vs MLL vs LLL, and MOL vs LOL) (**Table 1**).

**Table 1:** Differences in drought stress among the heteromorphic leaves.

Groups	Midday leaf water potential (MPa)	MDA content (μg·g^-1^)	Proline content (μg·g^-1^)	Soluble sugar content (μg·g^-1^)
SLL	-1.48 ± 0.26a	6.14 ± 0.42c	21.37 ± 2.97c	43.62 ± 4.50b
MLL	-1.25 ± 0.24a	5.93 ± 2.41c	21.63 ± 14.99c	42.70 ± 7.00b
LLL	-1.31 ± 0.10a	5.53 ± 1.81c	38.01 ± 6.93bc	37.82 ± 9.48b
MOL	-1.53 ± 0.24a	9.39 ± 0.80b	50.50 ± 6.25ab	69.57 ± 1.06a
LOL	-1.64 ± 0.21a	10.38 ± 0.94b	40.59 ± 14.18b	75.9 ± 10.42a
LBL	-2.10 ± 0.12b	13.62 ± 0.44a	65.54 ± 4.33a	76.8 ± 10.42a
*F*	6.56	16.87	9.67	21.19
*p*-values	*P*<0.05	*P<*0.01	*P<*0.01	*P<*0.01

SLL, MLL, LLL are lanceolate leaves distributed in the whole crown of the small trees, the bottom of the medium and large tree crowns, respectively. MOL and LOL are ovate leaves grown in the top of the medium tree crowns and the intermediate of the large tree crowns, respectively. LBL is the broad-ovate leaves only located at the top of the large tree crowns. F and p-values are the results of One-way ANOVA. Different letters on the back of the related variables indicate the significant differences among heteromorphic leaves, while the same letters show the insigniﬁcant difference. Values are represented as mean ± SD.

### 3.2 RNA-seq analysis of heteromorphic leaves

Eighteen cDNA libraries were constructed from total RNA. Sequencing data were first preprocessed to meet the quality control (QC) standards. The average reading after QC for SLL, MLL, LLL, MOL, LOL and LBL was 54636841, 54899515, 49631972, 56285399, 53465650 and 49995463, respectively. The basal percentages of Q20 and Q30 were above 95% and 88%, respectively, while the basal percentages of G and C ranged from 43% to 45%. These indicated that the sequencing results were qualified and reliable (**Supplementary Table S1**). According to analysis result of the genomic localization compared with the reference genome (*Populus tomentosa*), the total mapped reads accounted for more than 90% of the clean reads. The unique mapped reads were also greater than 80% (**Supplementary Table S2**). The correlation coefficient of gene expression among groups was closer to 1, suggesting that gene expression has higher similarity. Also, as showed from **Supplementary Figure S1**, *R* ^2^ between the replicates for each group is ≥0.8, indicating that the sample collection is reasonable.

**Figure 2:**
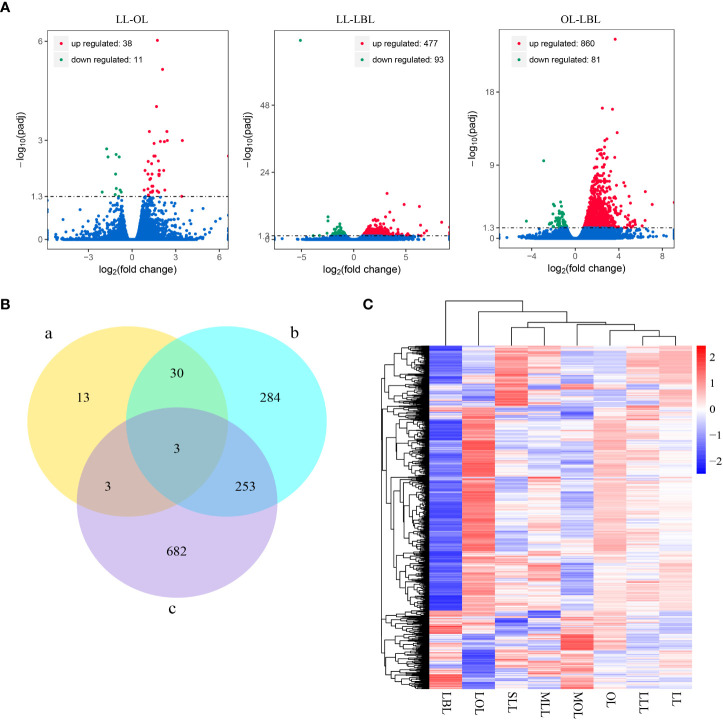
Difference in DEGs among three heteromorphic leaves. **(A)** Differential gene distribution. Red, green and blue dots indicated genes are up-regulated, down-regulated and no significant differential expression, respectively. **(B)** Number of DEGs in the pairwise group. The a-c indicate the lanceolate-ovate, lanceolate-broad-ovate, and ovate-broad-ovate DEGs pairwise group, respectively. **(C)** Overall FPKM hierarchical clustering heat map. Red and blue indicate the genes with high and low expression, respectively. The color change from red to blue indicates a change in the log_10_(FPKM+1) from large to small. LL, OL and LBL are lanceolate, ovate and broad-ovate leaves.

Results showed that drought stress was no significant difference between the same type of leaves (SLL vs MLL vs LLL; and MOL vs LOL), but significantly differed among the different types of heteromorphic leaves. This indicated that it was meaningless to analyze the differences of drought stress and genes in the same type of leaves. Thus, in order to analyze the differences in gene expression among different heteromorphic leaves, we classified six leaf groups into three categories: lanceolate leaf (included SLL, MLL and LLL); ovate leaf (composed of MOL and LOL) and broad-ovate leaf (LBL). To determine the total number and types of DEGs, we performed a quality control screen on the transcriptome data and selected DEGs with P-value<0.05 for further study. After DEGs analysis, the results found that a total of 49 DEGs were identified in lanceolate-ovate leaf pairs, including 38 up-regulated genes and 11 down-regulated genes. In terms of lanceolate-broad-ovate leaf pairs, a total of 570 DEGs were identified, including 477 were up-regulated DEGs and 93 down-regulated DEGs. For ovate-broad-ovate leaf pairs, 941 DEGs have been identified, which comprised of 860 up-regulate DEGs and 81 down-regulated DEGs (**Figure 2A**). The specific DEGs in lanceolate, ovate and broad-ovate leaves were 13, 284, and 682, respectively. The shared DEGs in the pairs of lanceolate and ovate leaves, of lanceolate and broad-ovate leaves, as well as of ovate and broad-ovate leaves were 6, 33 and 256, respectively. Notably, there were 3 shared DEGs among heteromorphic leaves, including the myb-related protein Myb4-like (LOC105142516), aquaporin TIP4-1 (LOC105122290) and hyoscyamine 6-dioxygenase-like (LOC105136845) (**Figure 2B**). All DEGs were perform clustered with according to gene expression patterns and were evaluated using log10(FPKM+1) (Fragments per kilobase per million mapped reads) of the two groups (**Figure 2C**).

GO enrichment analysis showed, compared with lanceolate leaves, 181 biological processes were upregulated and 191 were downregulated, while 37 cellular components were upregulated and 45 were downregulated, but 105 molecular functions were upregulated and 73 were downregulated in ovate leaves. Compared with broad-ovate leaves, 778 and 341 biological processes were upregulated and downregulated, while 152 and 122 cellular components were upregulated and downregulated, but 380 and 225 molecular functions were upregulated and downregulated in lanceolate leaves, respectively. And in broad-ovate leaves, compared with ovate leaves, 1006 biological processes were upregulated and 348 were downregulated, 229 cellular components were upregulated and 59 were downregulated, and 480 molecular functions were upregulated and 184 were downregulated (**Supplementary Table S3**). Additionally, our results showed the most representative cellular components were mainly concentrated in membranes, cells and organelles. The most representative molecular functions were related with catalytic, oxidoreductase, hydrolase, kinase and protein activities. Metabolic, macromolecular biosynthesis process, immune response activation, redox process, cell migration, template transcription regulation, immune response and cell wall organization were significantly enriched in the biological processes (**Supplementary Figure S2**).

### 3.3 Genes associated with the formation of heteromorphic leaves

We further analyzed the GO enrichment results and found that 36 differential expressed genes were associated with drought tolerance, plant growth and development. After considering that the occurrence of heteromorphic leaves was essentially a problem of leaf-shaped development, 7, 6 and 5 DEGs were found to be related to cell wall organization or biogenesis, cell wall modification and pectinesterase activity, respectively. In addition, 6, 6 and 7 DEGs of them were found to be associated with encoding 3-oxoacyl-[acyl-carrier-protein] synthase (ACP) activity, cell division, and photosynthesis and sucrose biosynthesis, respectively.

**Figure 3:**
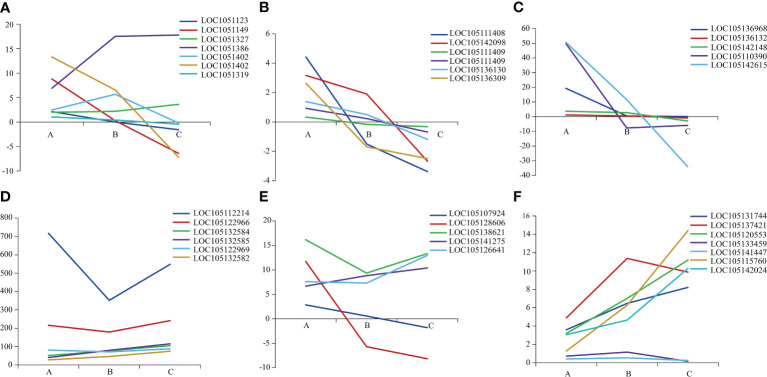
Expression difference in genes associated with plant growth and development among three heteromorphic leaves. A, B and C is the lanceolate, ovate and broad-ovate leaves, respectively. The Y-axis represents the difference between the expression (FPKM) of the corresponding genes among three heteromorphic leaves. **(A–F)** are genes related to cell wall organization or biogenesis, cell wall modification, pectinesterase activity, encoding ACP activity, cell division and photosynthesis, respectively.

The 36 DEGs associated with plant growth and development showed different expression among three heteromorphic leaves, which were mainly down-regulated in the ovate and broad-ovate leaves compared with lanceolate leaves (**Figure 3**). In terms of the genes related to cell wall organization or biogenesis, the expression of the fucosyltransferase (FUT, LOC105132716) and MADS-box protein (LOC105138623) in the broad-ovate leaves were up-regulated largely than lanceolate leaves, but the rest of genes were down-regulated (**Figure 3A**). For the genes related to cell wall modification and pectinesterase activity, our results showed the genes encoding pectin methylesterase (PME) and pectin methylesterase inhibitor (PMEI) were down-regulated largely in broad-ovate and lanceolate leaves compared with broad-ovate leaves (**Figures 3B, C**). The DEGs encoding ACP activity were all up-regulated in the ovate and broad-ovate leaves compared with lanceolate leaves (**Figure 3D**). Among the genes related to cell division, 2 DEGs of them were up-regulated highly in lanceolate than ovate and broad-ovate leaves, but the remaining 4 DEGs showed an opposite pattern (**Figure 3E**). The expressions of the genes encoding photosynthesis were differed among three heteromorphic leaves. The auxin-responsive protein (IAA, LOC105137421), LIM domain-containing protein WLIM1-like (CSRP, LOC105120553), sucrose phosphate synthase (SPS, LOC105115760), and sucrose synthase (SUS, LOC105142024) was up-regulated largely in ovate and broad-ovate leaves than lanceolate leaves. However, the cytochrome b561 (Cyt-b561, LOC105141447) and ABC transporter (LOC105133459) showed opposite pattern (**Figure 3F**).

### 3.4 KEGG pathway enrichment analysis of DEGs

**Figure 4:**
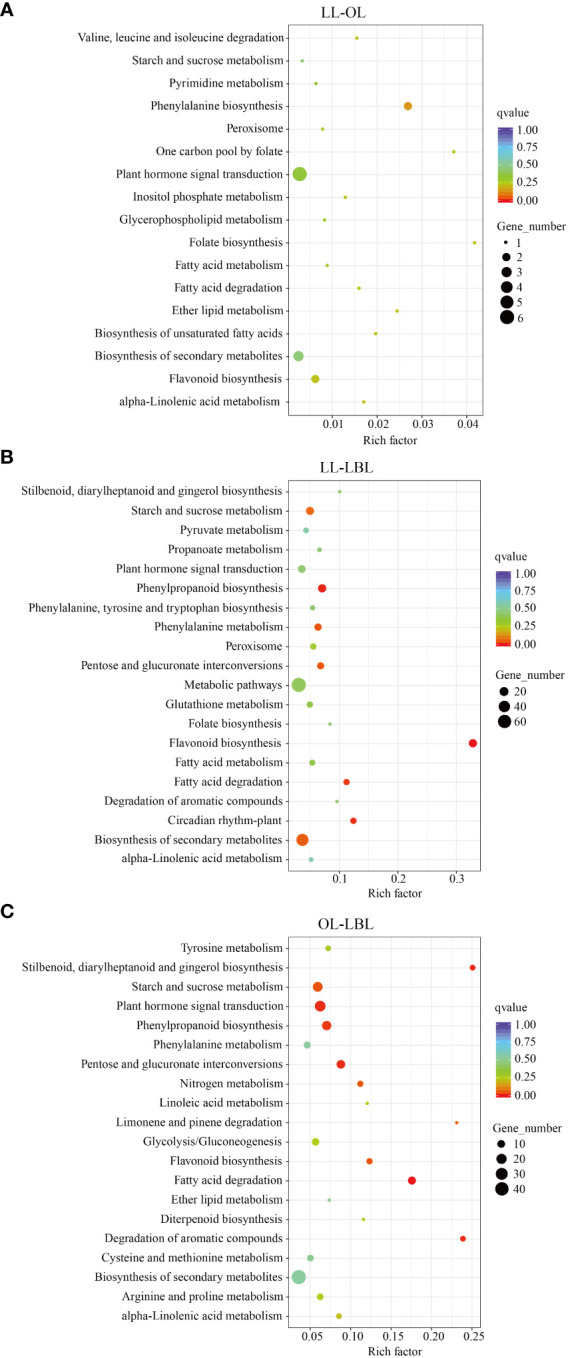
Statistics of KEGG pathway enrichment. High and low values are showed in red and green, respectively. **(A–C)** indicate the lanceolate-ovate, lanceolate-broad-ovate, and ovate-broad-ovate DEGs pairwise group, respectively.

The results of KEGG pathway enrichment analysis showed that, a total of 1560 DEGs were assigned to 81 KEGG pathways (**Supplementary Table S4**). In the pair between lanceolate and ovate leaves, four pathways were found to be enriched: phenylpropanoid biosynthesis (pop00940)), fatty acid degradation (pop00071), and peroxisome (pop04146) (**Figure 4A**). In the pair between lanceolate and broad-ovate leaves, 20 pathways were found to be enriched significantly, including flavonoid biosynthesis (pop00941), phenylpropanoid biosynthesis (pop00940), circadian rhythm-plant (pop04712) and fatty acid degradation (pop00071) (**Figure 4B**). In terms of the pair between ovate and broad-ovate leaves, the pathways associated with fatty acid degradation (pop00071), stilbenoid, diarylheptanoid and gingerol biosynthesis (pop00945), pentose and glucuronate interconversions (pop00040) and degradation of aromatic compounds (pop01220) were significantly enriched (**Figure 4C**). In order further to explore the formation mechanism of heteromorphic leaves, we compared the differences in KEGG pathways among three heteromorphic leaves. The results showed that the essential pathways related to phytohormone signal transduction and resistance response were successfully annotated from KEGG pathways of three heteromorphic leaves; which included phenylpropanoid biosynthesis (pop00940), flavonoid biosynthesis (pop00941), and plant hormone signal transduction (pop04075), and starch and sucrose metabolism (pop00500). They may have contributed to the change in leaf shape.

**Figure 5:**
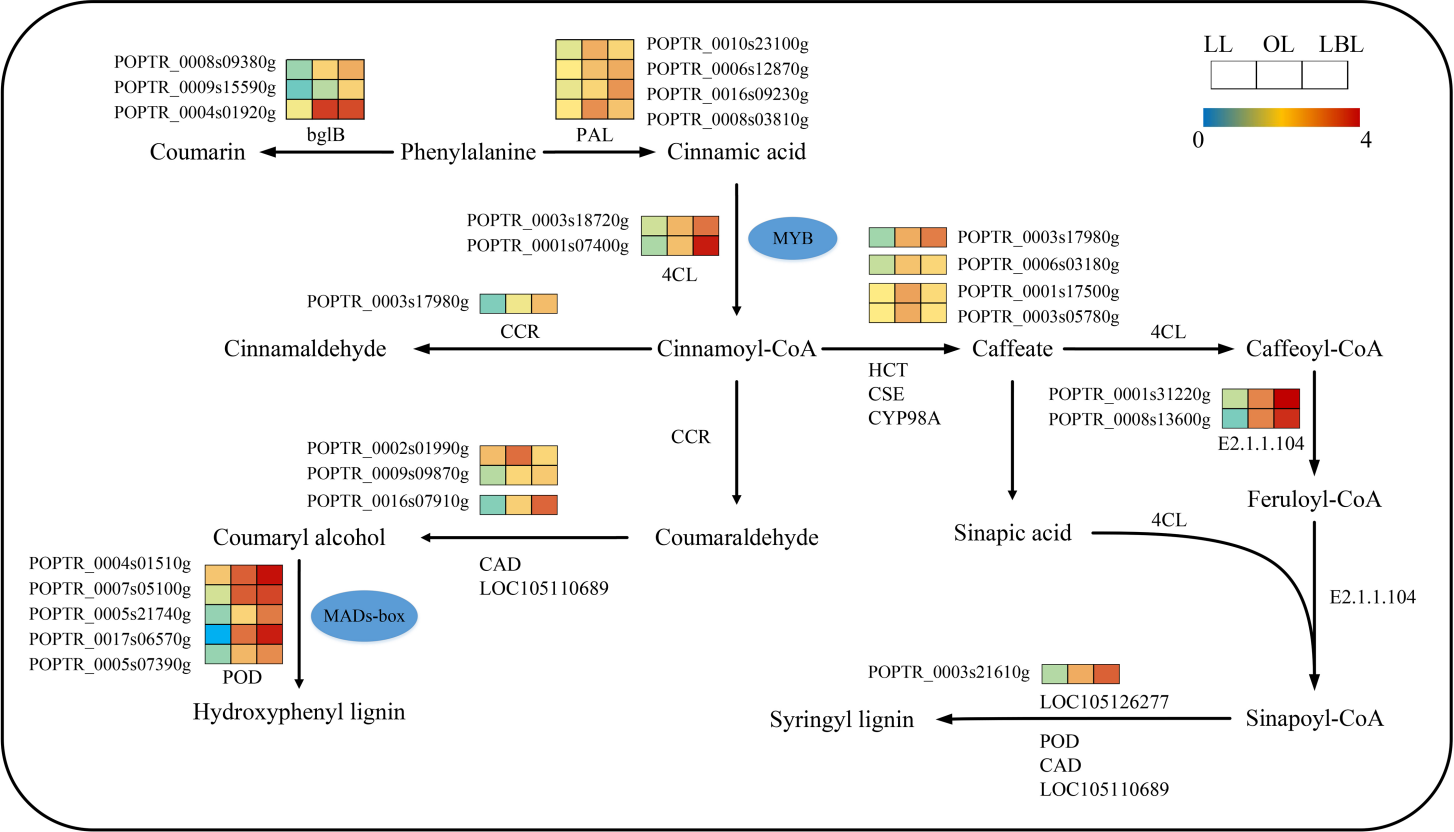
DEGs associated with the phenylpropane biosynthetic pathway in three heteromorphic leaves. The colored squares next to each gene are heat maps of the expression of key enzyme genes for the phenylpropane biosynthesis. LL, OL and LBL are lanceolate, ovate and broad-ovate leaves.

KEGG pathway enrichment analysis revealed that 232 transcripts were expressed in the phenylpropane biosynthetic pathway (**Figure 5**). Only nine DEGs were down-regulated in the lanceolate leaves, while all DEGs up-regulated in the ovate and broad-ovate leaves. The phenylpropane biosynthesis pathway is associated with the generation of a variety of phenylalanine derivatives, many of which can be used as precursors for synthesizing important compounds (flavonoids, coumarin, lignin, anthocyanins, tannins, etc.), and participate in the biosynthesis of lignin. Our results showed that the peroxidase (POD), LOC105110689, and LOC105126277 in the ovate and broad-ovate leaves were up-regulated during the synthesis of hydroxyphenyl lignin and syringyl lignin compared with the lanceolate leaves. The bglB and phenylalanine ammonia-lyase (PAL) was involved in the synthesis of coumarin and cinnamic acid, respectively. The shikimate O-hydroxycinnamoyltransferase (HCT), caffeoylshikimate esterase (CSE), and 5-O-(4-coumaroyl)-D-quinate 3’-monooxygenase (CYP98A) were involved in the synthesis of caffeate.

**Figure 6:**
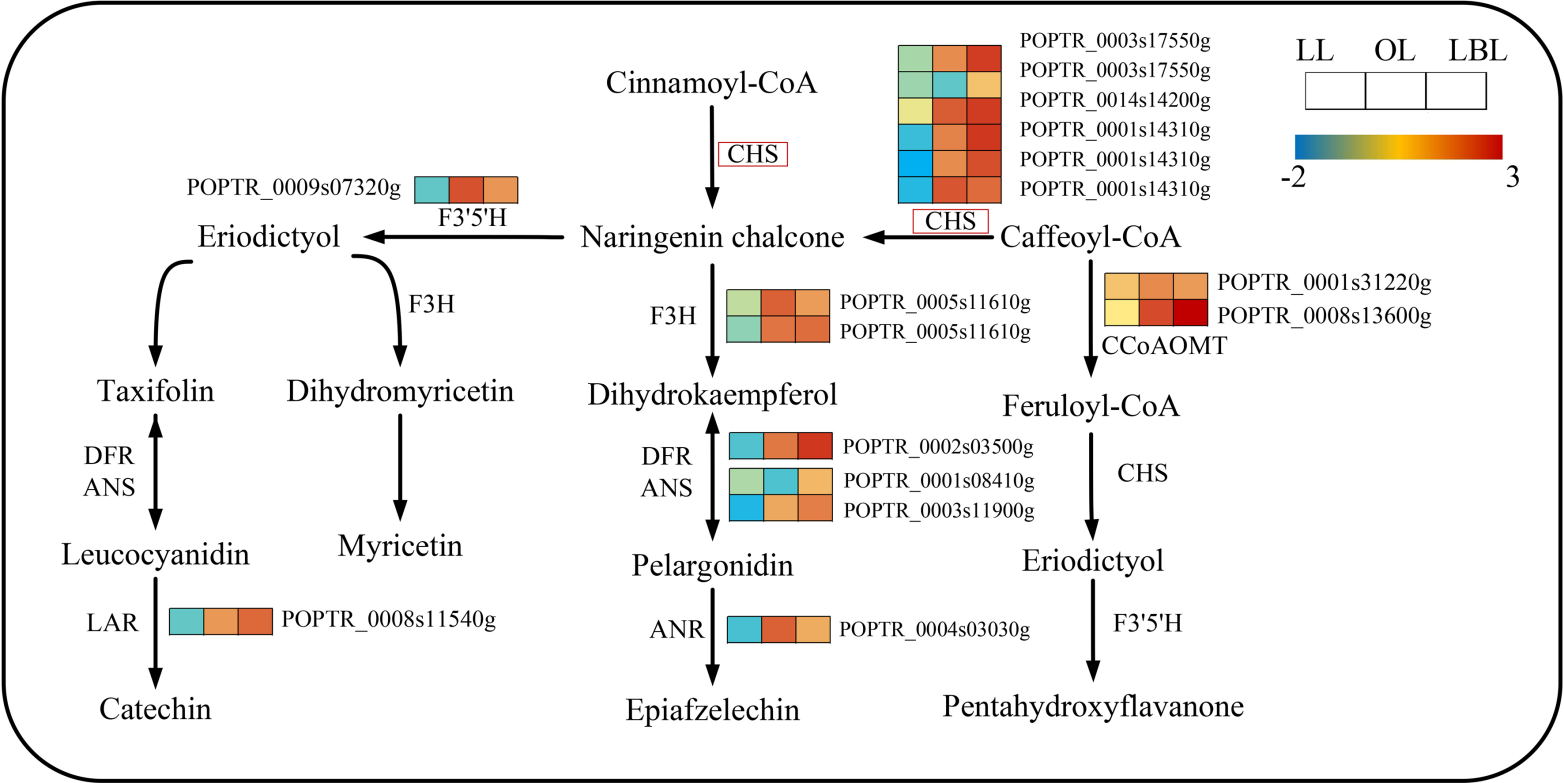
DEGs associated with the flavonoid biosynthesis pathway in three heteromorphic leaves. The colored squares next to each gene are heat maps of the expression of key enzyme genes for the flavonoid biosynthesis. LL, OL and LBL are lanceolate, ovate and broad-ovate leaves.

In terms of the flavonoid biosynthesis pathway, 16 and 3 DEGs were down-regulated in lanceolate and ovate leaves, whereas 16 and 19 DEGs up-regulated in ovate and broad-ovate leaves, respectively (**Figure 6**). The expression of 6 DEGs encoding chalcone synthase (CHS) were up-regulated largely in ovate and broad-ovate leaves than lanceolate leaves. Such change catalyzed the formation of chalcone (a key enzyme for activating flavonoid biosynthesis) from cinnamoyl co-A and caffeoyl co-A.

**Figure 7:**
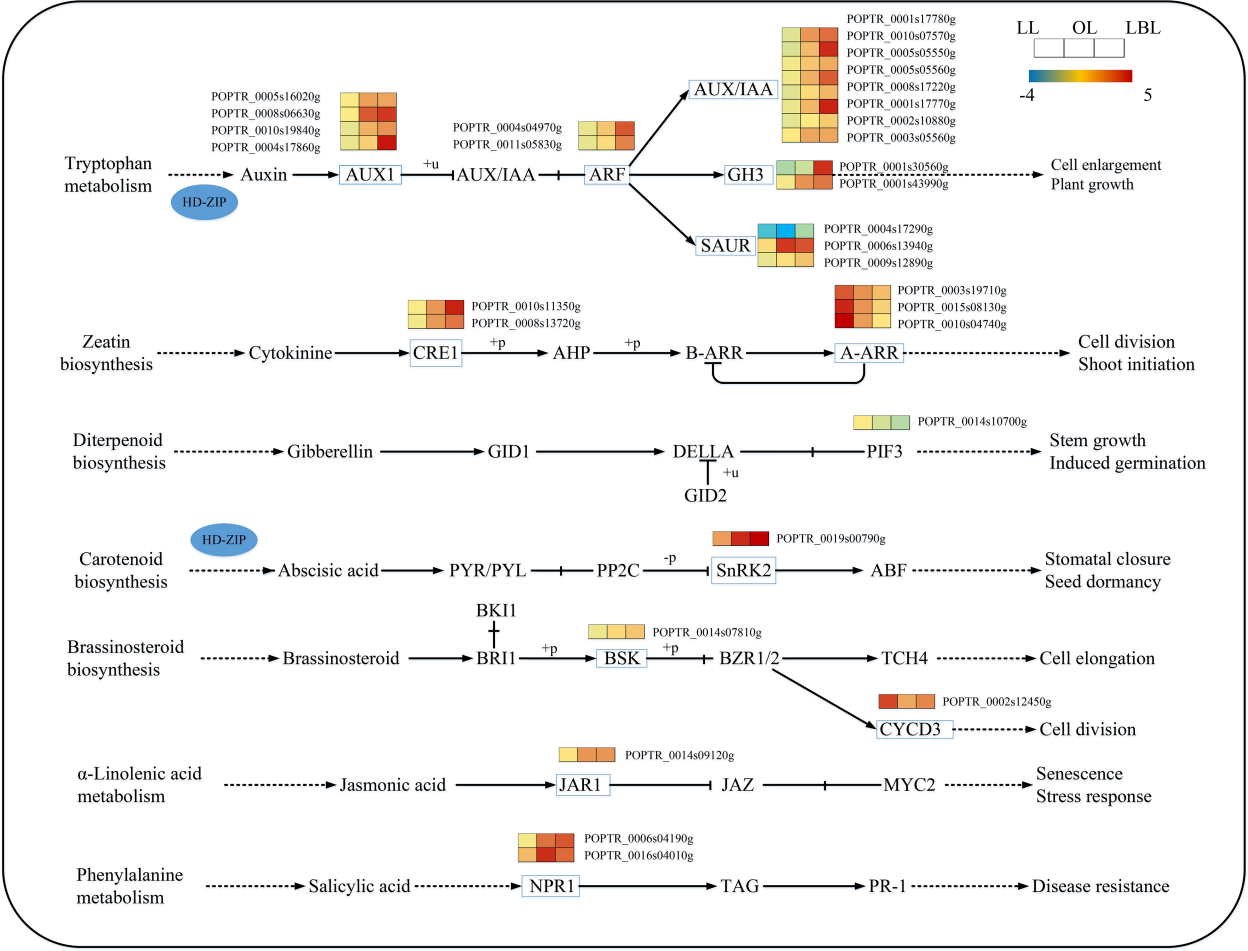
DEGs associated with the plant hormone signaling pathway in three heteromorphic leaves. The colored squares next to each gene are heat maps of the expression of key enzyme genes for the plant hormone signaling. LL, OL and LBL are lanceolate, ovate and broad-ovate leaves.

With regard to plant hormone signaling pathway, our results found that 16 and 3 DEGs were down-regulated in lanceolate and ovate leaves, while 9 and 22 DEGs were up-regulated, respectively. In the broad-ovate leaves, 2 DEGs were down-regulated, while the rest of DEGs were all up-regulate (**Figure 7**). Among the auxin biosynthesis genes, the expression of auxin influx carrier (AUX1) and auxin/indole-3-acetic acid (Aux/IAA) genes were up-regulated largely in the broad-ovate leaves than the other two leaves. One of the small auxin-up RNA (SAUR) genes was up-regulated while another was down-regulated. AUX1, AUX/IAA and Gretchen Hagen 3 (GH3) protein were most significantly expressed in the ovate and broad-ovate leaves than the lanceolate leaves. In terms of genes involved in the abscisic acid, jasmonic acid, and salicylic acid biosynthesis, the expressions in the ovate and broad-ovate leaves were all up-regulated highly than that in the lanceolate leaves. Also, a down-regulated gene of Sucrose non-fermenting1-related protein kinase 2 (SnRK2) involved in abscisic acid biosynthesis was recorded in the broad-ovate leaves. At the early stage of leaf development, cell division-related genes were significantly up-regulated in the lanceolate leaves, while the gene of phytochrome-interacting factor 3 (PIF3) involved in gibberellin biosynthesis was down-regulated in the broad-ovate leaves.

**Figure 8:**
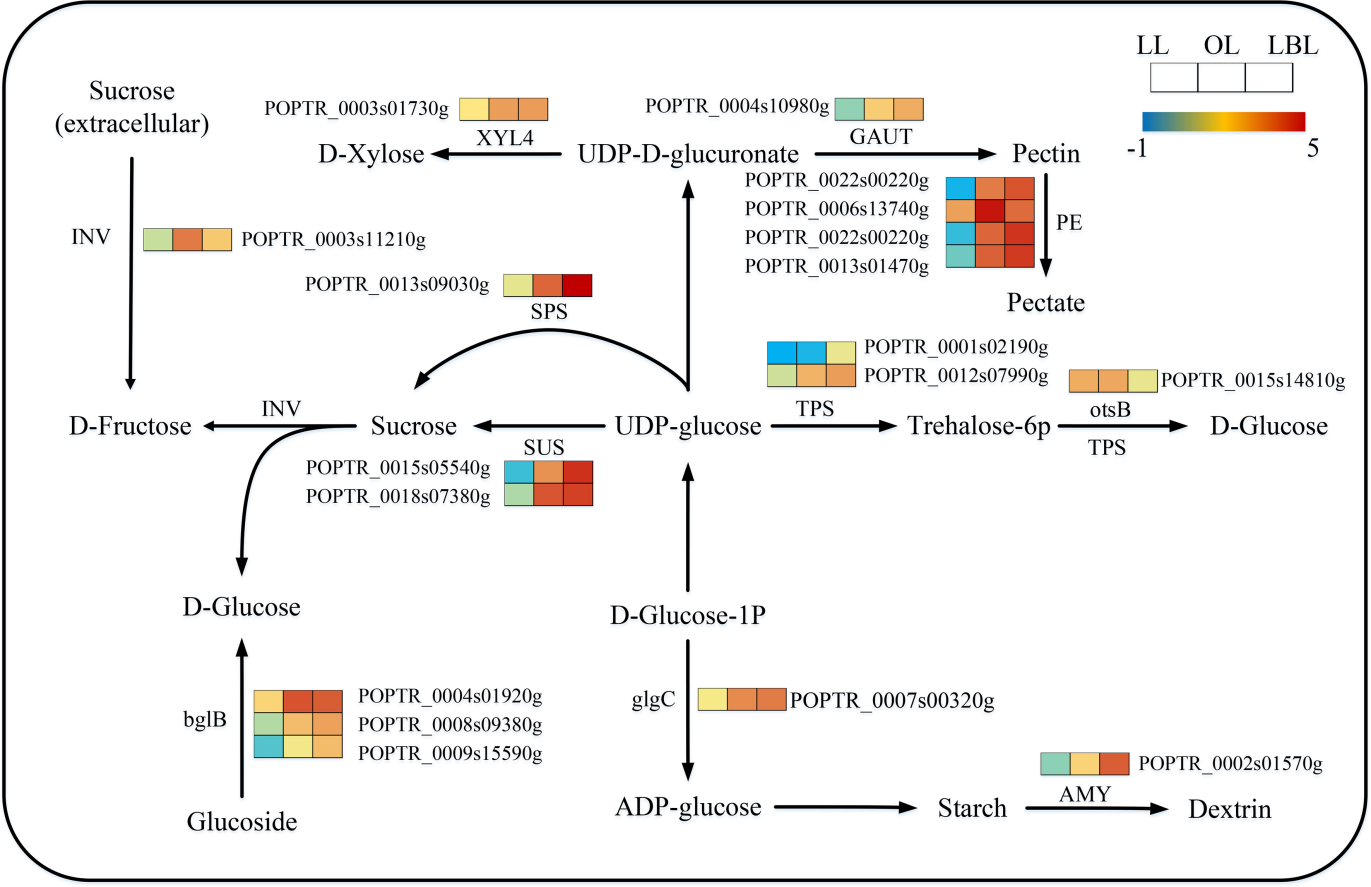
DEGs associated with the starch and sucrose metabolism pathway in three heteromorphic leaves. The colored squares next to each gene are heat maps of the expression of key enzyme genes for the starch and sucrose metabolism. LL, OL and LBL are lanceolate, ovate and broad-ovate leaves.

331 transcripts were identified to relate with the starch and sucrose metabolism. The expressions of these DEGs were different among the three heteromorphic leaves. 15 and 1 DEGs were down-regulated in the lanceolate and ovate leaves, respectively. Only 9 DEGs were up-regulated in the lanceolate leaves, however, all DEGs in the ovate and broad-ovate leaves were up-regulated (**Figure 8**). Starch and sucrose play the essential roles in plant organ growth and drought tolerance by acting as osmotic regulator and inducing the accumulation of many metabolites in leaves. Therefore, changes in the expression of genes associated with them may affect the formation of heteromorphic leaves. Our results showed that TPS, otsB, beta-glucosidase (bglB), and glucose-1-phosphate adenylyltransferase (glgC) were significantly up-regulated in the synthesis of D-glucose and ADP-glucose in the broad-ovate leaves compared with lanceolate and ovate leaves. The expressions of SPS, SUS, D-fructose (1), dextrin (1), and Xylose (1) genes were also up-regulated significantly in the broad-ovate leaves. Among all DEGs, Pectinesterase (PE) involving in pectin formation had the highest number of expressed transcripts (5 up-regulated).

## 4 Discussion

All woody plants in arid desert areas would suffer hydraulic constraints with the increase in height, especially arboreous plants (Wang et al., 2021). *P. euphratica* is the only tree species naturally distributed in arid desert communities (Yang et al., 2014). Because of severe local precipitation deficits, the plants are subject to long-term drought stress and are more hydraulic constraints than that other species (Keram et al., 2019). Due to the serious shortage of local precipitation, plants suffer from drought stress. With the increase in tree height, upper canopy leaves are exposed to the strongest solar radiation and evaporative demand (Nidhi et al., 2022), thus the water supply is more difficult than that of the lower leaves, which will cause more drought stress. For example, broad-ovate leaves located at the top of the large tree crowns showed lower midday leaf water potential and higher contents of MDA, proline, and soluble sugar. MDA. However, the same type of leaves located at the same height show similar adaptation strategies to drought stress because they are in similar environments and have consistent water supply capacity. Thus, there are no significant differences between leaves of the same type under drought stress.

Transcriptome analysis showed the expression patterns of different biosynthetic pathways varied the three heteromorphic leaves. According to the comparison of the heteromorphic leaves, our results showed that the number of up-regulation of DEGs was the highest in the pair between ovate and broad-ovate leaves (860), while the intermediate in the pair between lanceolate and broad-ovate leaves (477), but the lowest in the pair between lanceolate and ovate leaves (38). These might be involved in greater drought tolerance in broad-ovate leaves.

The role of the cell wall in controlling plant morphogenesis and regulating stress response has been demonstrated by a large number researcher (Chaudhary et al., 2021; Qiu et al., 2021). Arabinogalactan-proteins (AGPs) are essential proteoglycans for plant cell walls due to their involvement in cell elongation, expansion, and responses to biotic and abiotic stresses such as drought stress (Shi et al., 2003; Gaspar et al., 2004; Seifert and Roberts, 2007). In this study, the results found that the FUT gene encoding AGPs was up-regulated in three heteromorphic leaves. Additionally, the AGP content reached the highest level in broad-ovate leaves compared with lanceolate and ovate leaves. As a regulators of reactive oxygen species (ROS) homeostasis, OsMADs26 regulates many genes related to environmental stress, such as the regulation of plant synthesis of peroxidase, oxidoreductase, and NADPH-oxidase to increase the ability of resistance to environmental stress (Lee et al., 2008). In this study, the expression levels of MADs-box genes and peroxidase (POD) involved in lignin biosynthesis increased from the lanceolate to ovate to broad-ovate leaves, indicating that MAD can positively regulate the expression of POD to improve cellular antioxidant capacity, scavenge ROS, and protect the plant from drought stress. In addition, some MYB family members are also involved in the transcriptional regulation of xylogenesis and lignin biological processes (Geng et al., 2020; Chen et al., 2021). For example, researchers demonstrated that LmMYB15 can bind and activate the promoters of 4CL (Tang et al., 2021), which then as an important step to control lignin formation. In this study, our results found that three genes related to MYB transcription were co-expressed in all heteromorphic leaves, especially two 4CL transcripts were up-regulated in ovate to broad-ovate leaves, respectively. This suggests that the MYB family can promote the phenylpropane biosynthesis in heteromorphic leaves by regulating the expression of 4CL.This process suggests that the phenylpropane biosynthesis pathway, as an important component of plant resistance to environmental stress and other processes, may play a role in the response of broad-ovate leaves of *P. euphratica* to drought stress.

Previous studies have suggested that ACP may be involved in various biological processes in response to environmental stress (de Silva et al., 1990). For example, the expression of SbACP9 is up-regulated within 6-24 hours of water-deficient treatment in rice, showing a rapid and sustained response to drought stress (Ge et al., 2022). In this study, ACP enzymes exhibited the promoting activity in all three heteromorphic leaves of P. euphoretic, indicating that ACP may play an important role in attenuating the effects of abiotic stress through leaf morphological changes. In addition, the gene CHS encoding ACP activity also showed different up-regulated expression among the three heteromorphic leaves. Studies have pointed out that the flavonoid content increases when plants are exposed to a wide range of environmental stresses (Roberts and Paul, 2006). The main reason is that plants preferentially accumulate flavonoids with antioxidant properties, i.e., o-dihydroxy B-ring substituted quercetin and luteolin glycosides, to effectively inhibit the generation of free radicals and scavenge reactive oxygen species under abiotic stress (Brown et al., 1998; Massimiliano et al., 2004; Agati et al., 2011). Based on this scenario, we speculate that to adapt to increasing drought stress, the up-regulated expression of the CHS gene activate the biosynthesis of flavonoids and the accumulation of quercetin and luteolin glycosides in mesophyll cells. As the antioxidants, flavonoids are a crucial step in controlling cell growth and resisting stress with the change in leaf shape from lanceolate to ovate to broad-ovate.

It has been reported that plant hormones, especially AUX and ABA, can increase plant adaptation to various biotic and abiotic stresses by regulating the development of tissues and organs (Farooq et al., 2009; Du et al., 2012; Nakayama et al., 2017). In this study, two DEGs co-encoding the Homeo-Leucine Zipper ATHB (HD-ZIP) protein were gradually up-regulated from lanceolate to broad-ovate leaves. On the one hand, high expression of HD-ZIP protein leads to changes in plant morphology and growth, including embryonic, cotyledon, and leaf development (Hanson et al., 2001; Turchi et al., 2013). This has been demonstrated in Arabidopsis thaliana, where ATHB2 regulates plant development by regulating the auxin pathway. On the other hand, the high expression of ATHB can regulate the plant drought resistance by activating the expression of key genes in ROS signaling pathway and ABA-dependent pathway (Jiao et al., 2022). This study showed that the low expression of HD-ZIP and GH3 inhibit auxin synthesis in the lanceolate leaves. In contrast, high expression of Aux/IAA and auxin response factor (ARF) promotes the accumulation of auxin in the ovate and broad-ovate leaves. High expression of SNF1-related protein kinase 2 (SnRK2) activates the expression of ABA pathway. These results indicate that HD-ZIP has a potential role in linking AUX and ABA pathways, which makes broad-ovate leaves more resilient to drought stress than lanceolate leaves. Moreover, the major expression of SnRK2 has been found in hormone signaling pathways, indicating that SNRK2 might be a key gene in regulating the formation of heteromorphic leaves.

Photosynthesis is closely related to plant growth and drought tolerance because it determines the synthesis of organic matter and the exchange of water between leaves and atmosphere (Sela et al., 2020). For plants, adequate accumulation of organic matter in the body helps them resist drought stress (Brodribb and Hill, 1997), because plants tend to close stomata and weaken photosynthesis under the extreme drought stress (Koch et al., 2004). After the reduction of organic matter synthesized by photosynthesis, plants will use the non-structural sugars accumulated in the body, especially starch and sucrose, to maintain physiological activities (Trifilò et al., 2017). In addition, as osmotic substances, sugar content is related to cell permeability, which determines the water absorption capacity of roots and the ability of leaves to exchange water with the atmosphere (Du et al., 2020). The physiological change of photosynthesis has been confirmed in numerous studies. For example, the starch and sucrose contents of oak leaves grown in different positions are differed significantly (Alaoui-Sossé et al., 1996; Zhu et al., 2018). Sugar content is larger in broad-ovate leaves than lanceolate and ovate leaves of P. euphoretic (Yang et al., 2022b). In our study, we found that the expression of SPS and SUS in starch and sucrose pathways increased from lanceolate to ovate to broad-ovate leaves. This may have allowed the broad-ovate leaves to synthesize more starch and sucrose than the other two types of leaves. Therefore, the variable of gene expression related to photosynthesis process is beneficial to increase the ability of leaves to resist drought stress.

In this study, by measuring the indicators of drought stress in the heteromorphic leaves of *P. euphratica* (i.e., the midday leaf water potential and the contents of MDA, proline, and soluble sugar), we found that the broad-ovate leaves are all distinct from ovate and lanceolate leaves. 36 DEGs associated with the growth and development of the three heteromorphic leaves were screened. These DEGs might respond to drought tolerance by participating in phenylpropane biosynthetic, flavonoid biosynthesis, plant hormone signaling, and starch and sucrose metabolism, such as MADs-box, MYB, 4CL, CHS, and HD-ZIP. On this basis, we suggest that these key pathways of resistance to environmental stresses coordinate the simultaneous expression of DEGs of leaf shape development, thus inducing the formation of heteromorphic leaves of *P. euphratica*. These results provide new insights into the formation of heteromorphic leaves of *P. euphratica* and a theoretical basis for desert plants to adapt to drought stress. In addition, the transcriptome analysis in this study mainly serves as a prediction for further research and a technology for preliminary screening of potential candidate genes involved in inducing heteromorphic leaves, so future validations were also important.

## Data availability statement

The data presented in the study are deposited in the SRA repository, accession number PRJNA974730.

## Author contributions

All authors designed the study. Conceptualization, data curation, formal analysis, investigation, software, validation, visualization, and writing-original draft, RX; conceptualization, funding acquisition, investigation, writing, review, and editing, W-GL and X-DY; software, visualization and supervision, T-WH, B-RL, and H-XD. All authors contributed to the article and approved the submitted version.
