# Supplementary material for: Drought stress-induced the formation of heteromorphic leaves of Populus euphratica Oliv: evidence from gene transcriptome
Source: Front Plant Sci. 2023 Jun 7;14:1194169. doi: 10.3389/fpls.2023.1194169 (PMC10282185; doi:10.3389/fpls.2023.1194169)
Supplement: Supplementary file 1 [file DataSheet_1.docx]

Supplementary Material

Drought stress-induced the formation of heteromorphic leaves of *Populus euphratica* Oliv: evidence from gene transcriptome

Rui Xu, Wei-Guo Liu^*^, Ting-Wen Huang, Bo-Rui Li, Hui-Xian Dai^1^, Xiao-Dong Yang^*^

*** Correspondence:**Wei-Guo Liu, [wgliuxj@xju.edu.cn](mailto:wgliuxj@xju.edu.cn)
Xiao-Dong Yang, [xjyangxd@sina.com](mailto:xjyangxd@sina.com)

# Supplementary Tables and Figures

## Supplementary Tables

**Supplementary Table S1.** Transcriptome sequencing data statistics table.

**Supplementary Table S2.** Reads vs. reference genome comparison table.

**Supplementary Table S3.** GO enrichment list for differentially expressed genes.

**Supplementary Table S4.** Differential genes KEGG enrichment analysis.

##
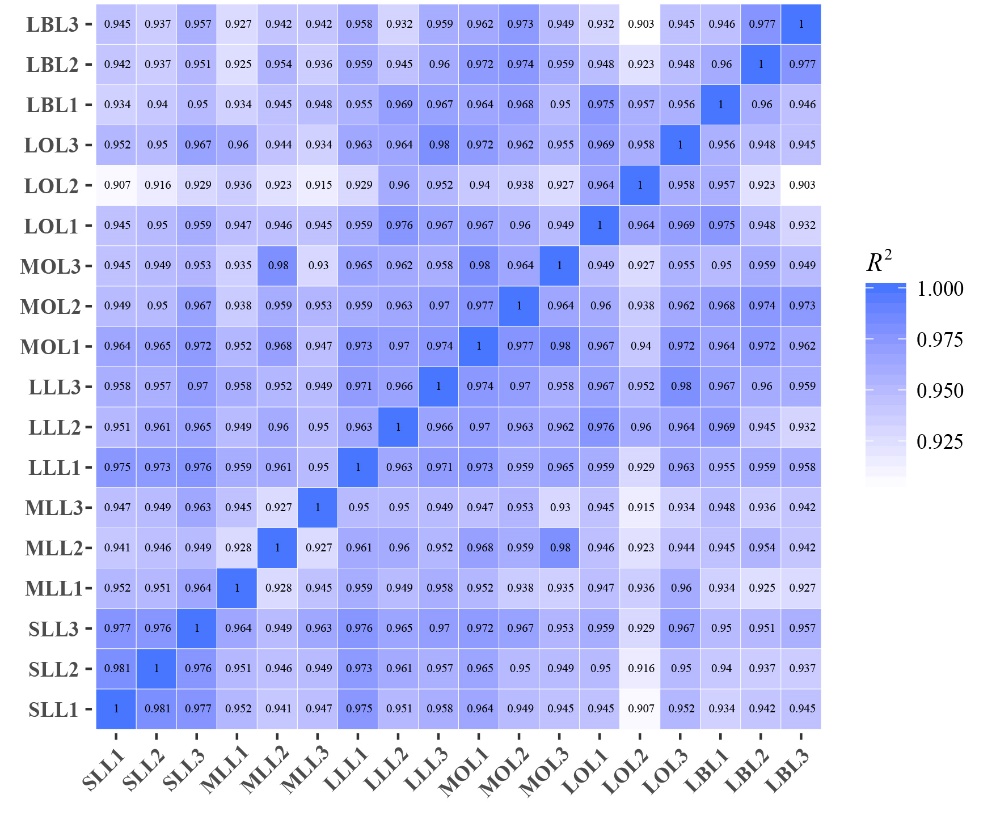
Supplementary Figures

**Supplementary Figure S1.** Correlation coefficient of gene expression between samples.


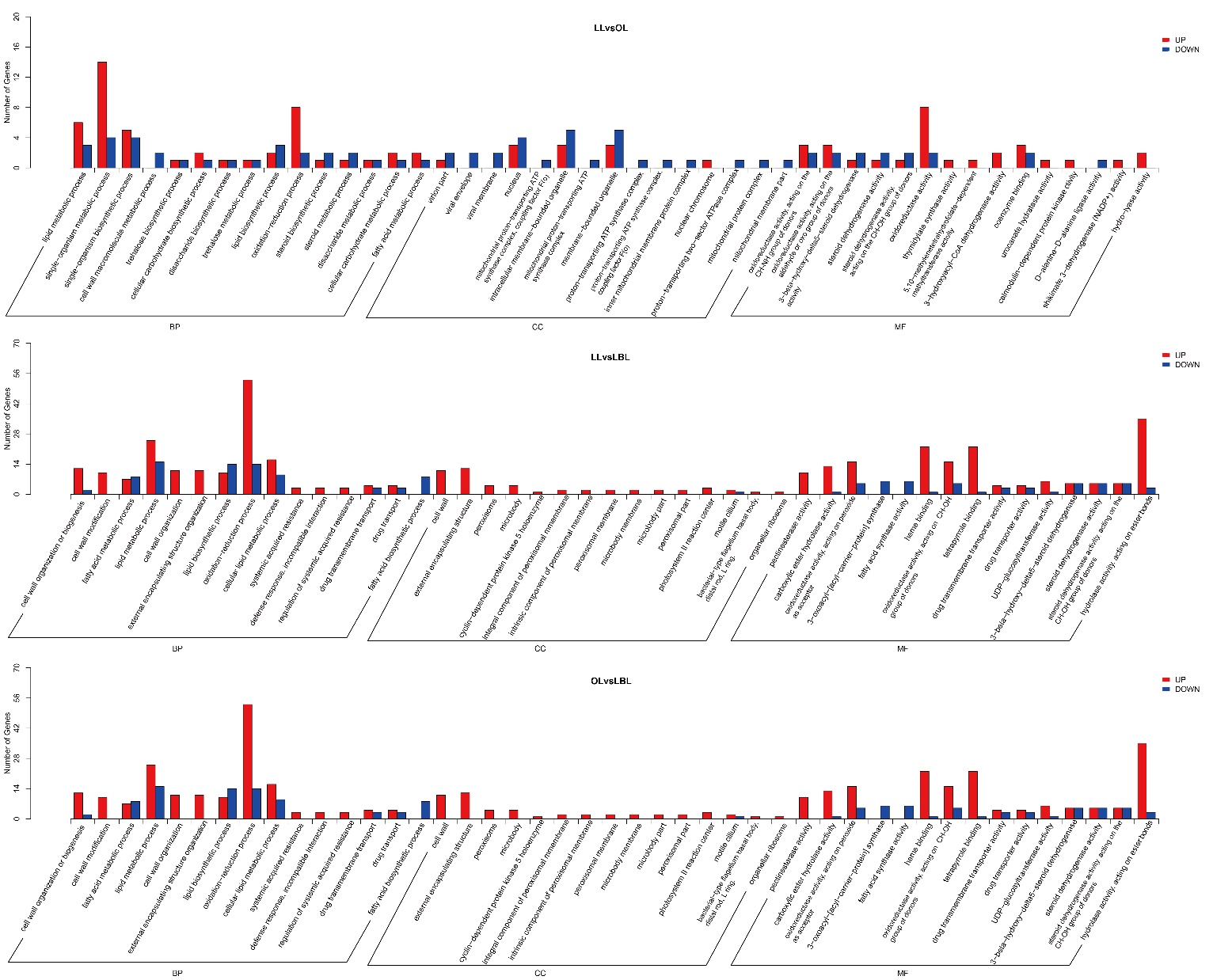
**Supplementary Figure S2.** GO distribution of DEGs among three heteromorphic leaves. GO categories were grouped into three levels: biological process, cellular component, and molecular function. The x- and y-axis indicates specific categories and the number of genes, respectively.
